# Cultivar-specific responses of the citrus endophytic microbiome to *Xanthomonas citri* subsp. *citri* infection reveals *Lysobacter* as a key biocontrol taxon

**DOI:** 10.3389/fpls.2025.1700610

**Published:** 2025-12-09

**Authors:** Yu Zhang, Waqar Ahmed, Zhenlin Dai, Han Meng, Hongmei Li, Ihab M. Moussa, Yonglin Ma, Jinhao Zhang, Guanghai Ji

**Affiliations:** 1 State Key Laboratory for Conservation and Utilization of Bio-Resources in Yunnan/Key Laboratory of Agro-Biodiversity and Pest Management of Ministry of Education, Yunnan Agricultural University, Kunming, Yunnan, China; 2 Plant Protection Research Institute, Guangxi Academy of Agricultural Science/Key Laboratory of Green Prevention and Control on Fruits and Vegetables in South China, Ministry of Agriculture and Rural Affairs/Guangxi Key Laboratory of Biology for Crop Diseases and Insect Pests, Nanning, Guangxi, China; 3 School of Breeding and Multiplication (Sanya Institute of Breeding and Multiplication), Hainan University, Sanya, China; 4 Center of Excellence in Biotechnology Research (CEBR), DSR, King Saud University, Riyadh, Saudi Arabia

**Keywords:** citrus canker, *Xanthomonas citri* subsp. *citri*, endophytic microbiome, biocontrol agents, Lysobacter antibioticus, citrus resistance

## Abstract

**Introduction:**

Citrus canker, caused by *Xanthomonas citri* subsp. *citri* (*Xcc*), is a major threat to citrus production worldwide, resulting in significant losses in yield and fruit quality. This study investigates the differential responses of endophytic microbial communities to *Xcc* infection in citrus cultivars with distinct resistance levels, specifically comparing the highly susceptible *Citrus reticulata* cv. ‘Orah’ and the more resistant *Fortunella crassifolia* cv. ‘Cuimi’. Through high-throughput amplicon sequencing, we characterized the bacterial and fungal communities in both cultivars before and after *Xcc* inoculation.

**Results:**

The results revealed distinct shifts in microbial diversity, with bacterial community diversity largely maintained in resistant cultivars but significantly reduced in susceptible ones following *Xcc* infection. Conversely, fungal community richness decreased in both cultivars post-inoculation, with notable cultivar-specific changes in the relative abundance of key genera. Notably, *Lysobacter* emerged as the only bacterial genus that significantly increased in abundance in the resistant cultivar under pathogen pressure, highlighting its potential as a key biocontrol agent. Further, we identified several fungal genera, including *Penicillium* and *Aspergillus*, which proliferated in susceptible plants under pathogen pressure. The study also isolated and identified a *Lysobacter antibioticus* GJ-6 strain with potent antagonistic activity against *Xcc*, offering insights into its potential role in enhancing disease resistance.

**Conclusions:**

This work provides a comprehensive understanding of how endophytic microbiomes differ between resistant and susceptible citrus cultivars, suggesting new avenues for developing sustainable biocontrol strategies to manage citrus canker. These findings underscore the potential of endophytes in mitigating plant diseases and advancing the application of microbiome-based interventions in agriculture.

## 1 Introduction

Citrus species are cultivated in more than 140 temperate and tropical countries worldwide. These economically vital crops are prized for their nutritional value, significant role in global trade, and diverse industrial applications across multiple sectors (Ma et al., 2020; Wang et al., 2022; Li et al., 2023). The citrus industry faces significant challenges from disease pressures, particularly citrus canker (*Xanthomonas citri* subsp. *citri*; *Xcc*), which has caused substantial production losses worldwide in recent years. This bacterial pathogen reduces fruit quality and yield while increasing management costs, creating economic burdens across the citrus value chain. The disease is caused by distinct pathotypes of *Xanthomonas*, with type A (*X. citri* subsp. *citri*) being the most aggressive, widespread, and economically damaging (Schaad et al., 2006). This pathotype, which is the only strain present in China, affects nearly all commercial citrus cultivars; however, the severity of the disease varies significantly among them. The pathogen affects multiple plant tissues, causing characteristic canker lesions on leaves, fruits, and stems. Severe infections lead to twig dieback, premature leaf and fruit abscission, and overall tree decline, ultimately resulting in significant yield reduction and quality deterioration (Ference et al., 2018).

Government statistics indicate that Guangxi, China, maintains 650,000 hectares of citrus cultivation, producing 1.9 billion kilograms annually, which accounts for 30% of the national output and 10% of global production as of 2023. Among the region’s key cultivars, ‘Orah’ mandarin (*Citrus reticulata* Blanco cv. ‘Orah’), a Temple × Dancy orange hybrid, ranks as one of China’s four major citrus varieties (Huang et al., 2022). However, this cultivar exhibits extreme susceptibility to citrus canker disease, often suffering severe yield losses and quality degradation upon *Xcc* infection (Qiu et al., 2022). In contrast, ‘Cuimi’ kumquat (*Fortunella crassifolia* Swingle cv. ‘Cuimi’), a high-value bud mutant of ‘Rongan’ kumquat indigenous to Guangxi (Liu et al., 2021b), displays notable tolerance. Although *Xcc* can infect all commercial citrus varieties, disease progression varies significantly among cultivars, with ‘Cuimi’ representing a rare example of partial resistance (Khalaf et al., 2011; Giraldo – González et al., 2021).

Currently, there is no complete cure for citrus canker disease. Disease control primarily relies on integrated strategies including: (1) strict quarantine measures to prevent pathogen spread, (2) removal and destruction of infected plant material, (3) cultivation of tolerant varieties, (4) chemical treatments, and (5) biological control methods (Naqvi et al., 2022). In commercial production, chemical pesticides remain the dominant control approach (Riera et al., 2018). Copper-based bactericides are widely used against various bacterial pathogens (Cervantes and Gutierrez-Corona, 1994; Izadiyan and Taghavi, 2024), while antibiotics such as streptomycin are also frequently employed (Stockwell and Duffy, 2012). Although judicious pesticide use can enhance plant disease prevention, overreliance on chemical controls poses significant risks. These include the emergence of resistant bacterial strains, phytotoxic effects on fruit surfaces, and environmental pollution (Graham et al., 2008; Zhang et al., 2003; Behlau et al., 2020). Biocontrol methods for citrus canker have recently gained significant attention as an environmentally sustainable alternative to chemical treatments. These approaches offer multiple advantages, including effective disease suppression, reduced production costs, and minimized risk of pathogen resistance development (Qian et al., 2021; Ke et al., 2023).

However, critical challenges remain in achieving stable colonization and maintaining the long-term efficacy of biocontrol agents (Bonaterra et al., 2022), as identifying antagonistic microorganisms capable of thriving on the leaves of mature citrus trees has proven to be difficult (Ali et al., 2023). Endophytes, microorganisms residing within plant tissues without causing disease, are recognized as key players in plant health and pathogen resistance (Berg, 2009; Ali et al., 2023). Advances in high-throughput sequencing now allow for detailed profiling of these communities (Ahmed et al., 2025). However, the role of the entire endophytic microbiome (both bacteria and fungi) in conferring natural resistance to citrus canker remains poorly understood. While preliminary studies have identified differences in bacterial communities between asymptomatic and infected leaves (Adeleke et al., 2022; Huang et al., 2023) or noted *Bacillus subtilis* as a conserved species (Liu et al., 2021a), a controlled, comparative investigation of the microbiome in genetically resistant versus susceptible cultivars is lacking. Such a study is crucial for identifying microbial taxa and consortia directly associated with the host’s resistance phenotype.

Given the complex interplay of factors influencing plant endophytic communities, we hypothesized that the resistant cultivar ‘Cuimi’ would harbor a more resilient and responsive endophytic microbiome, which would undergo distinct, beneficial structural shifts upon *Xcc* challenge, potentially enriching for antagonistic bacteria. To test this, we employed a controlled experimental design using the susceptible ‘Orah’ and tolerant ‘Cuimi’ cultivars under uniform conditions. We systematically inoculated both cultivars with *Xcc* to: (1) characterize dynamic changes in leaf endophytic microbial communities pre- and post-inoculation, (2) elucidate differential responses of bacterial and fungal communities to pathogen challenge, and (3) furthermore, we aimed to isolate and identify key microbial taxa associated with the resistance phenotype, thus bridging correlation with function and providing a framework for developing targeted biocontrol strategies against citrus canke.

## 2 Materials and methods

### 2.1 Plant material

One-year-old citrus seedlings were used in this study, including *Citrus reticulata* cv. ‘Orah’ (extremely susceptible cultivar) and *Fortunella crassifolia* cv. ‘Cuimi’ (highly tolerant cultivar). Plants were maintained in 30 × 30 cm pots containing 10 kg of soil at the experimental base of the Institute of Plant Protection, Guangxi Academy of Agricultural Sciences, Nanning, China (22°50’57”N, 108°14’38”E). Three weeks before experimentation, plants were pruned to synchronize young leaf development. During June 2024, plants were maintained under controlled greenhouse conditions: temperature, 28°C ± 2°C; relative humidity, 80% ± 10%; and photoperiod, 14 h light/10 h dark (Giraldo – González et al., 2021).

### 2.2 Bacterial strain and culture conditions

The experiment utilized strain YSPT, a highly virulent *Xanthomonas citri* subsp. *citri* (*Xcc*) isolated from Guangxi, which demonstrates greater virulence than the reference strain *Xcc* 306 (de Oliveira et al., 2016). The strain was maintained in nutrient broth (NB) medium or on nutrient agar (NA) plates (tryptone 5 g/L, sucrose 10 g/L, beef extract 3 g/L, yeast extract 1 g/L, and pH 7.0; for NA, agar 15 g/L) at 28°C (Teper et al., 2020). Liquid cultures were initiated from isolated colonies on plates and grown under shaking (160 rpm) for 24 h. After growth, the cultures were adjusted to an optical density (OD_600nm)_ of 0.6 (10^8^ CFU/mL) using sterile distilled water, as measured with the aid of a spectrophotometer (Saldanha et al., 2022).

### 2.3 Inoculation of bacterial strain, experimental conditions, and symptoms observation

Leaf inoculation was performed using a modified hand-held syringe infiltration method. For each treatment, 10 µL of either sterile NB or *Xcc* suspension (OD_600nm_ = 0.6) was slowly infiltrated by pressing a needleless syringe tip against the abaxial leaf surface while applying gentle counter-pressure with a finger on the opposite side, creating a characteristic water-soaked infiltration zone. Three infiltration sites were established per leaf, one on each side of the midvein. Four experimental treatments were implemented: (1) WGC (Orah + NB control), (2) JJC (Cuimi + NB control), (3) WGT (Orah + *Xcc*), and (4) JJT (Cuimi + *Xcc*). The experimental design consisted of three biological replicates per treatment, with three plants per replicate (totaling 36 plants) and eight inoculated leaves per plant, maintaining consistent developmental stages across all samples. Disease symptoms were monitored daily following inoculation. At three days post-inoculation (3-dpi), which was determined to be the point where symptoms were fully developed and clearly distinguishable between cultivars, representative leaves from each plant and treatment were photographed.

### 2.4 Sample collection

Leaf samples were collected at 3-dpi when characteristic disease symptoms became apparent. This time point was selected for microbiome analysis to capture the early, dynamic shifts in the endophytic communities in direct response to pathogen challenge, before extensive tissue necrosis could irreversibly alter the microbial niche. For sequencing, four symptomatic leaves from each plant were harvested and pooled to form a single composite sample per biological replicate. These pooled samples were then subjected to a rigorous surface sterilization protocol: initial 30-second rinse in sterile distilled water, followed by sequential immersion in 70% ethanol (30 seconds), 2.5% NaClO solution containing 0.1% Tween 80 (30 seconds), and a final 70% ethanol wash (15 seconds). Between each sterilization step, leaves were thoroughly rinsed three times with sterile distilled water to remove residual disinfectants. The efficacy of the surface sterilization was validated by plating 100 µL of the final sterile distilled water rinse onto NA and potato dextrose agar (PDA) plates. No microbial growth was observed after 48–72 hours of incubation at 28°C, confirming the removal of surface epiphytes. Using sterile scissors, petioles were aseptically trimmed before placing the leaves in pre-labeled screw-cap tubes. The samples were immediately flash-frozen in liquid nitrogen and stored at -80°C to preserve nucleic acid integrity until DNA extraction was performed. The remaining symptomatic leaves from different treatments were collected, labeled accordingly, and directly stored at 4°C for endophyte isolation.

### 2.5 DNA extraction and amplicon sequencing

Total genomic DNA was extracted from surface-sterilized leaf samples using the E.Z.N.A.^®^ Plant DNA isolation kit (Omega Bio-Tek) according to the manufacturer’s instructions. DNA quality was assessed through dual approaches: concentration and purity (A260/A280 ratio) were measured using a NanoDrop spectrophotometer (Thermo Scientific), while integrity was verified by electrophoresis on 1% agarose gels (Li et al., 2025). For bacterial community analysis, the V5-V7 hypervariable regions of the 16S rRNA gene were amplified using barcoded primers 799F (5’-barcode-AACMGGATTAGATACCCKG-3’) and 1193R (5’-ACGTCATCCCCACCTTCC-3’) (Liu et al., 2020). Fungal communities were characterized by amplifying the ITS1 region with primers ITS1F (5’-barcode-CTTGGTCATTTAGAGGAAGTAA-3’) and ITS1R (5’-GCTGCGTTCTTCATCGATGC-3’) (Liang et al., 2020). PCR amplification was performed in triplicate 20 μL reactions containing: 4 μL 5× FastPfu Buffer, 2 μL 2.5 mM dNTPs, 0.8 μL each primer (5 μM), 0.4 μL FastPfu Polymerase, and 10 ng template DNA. The thermal cycling protocol consisted of an initial denaturation step at 95°C for 5 min, followed by 29 cycles of 95°C for 30 s, 55°C for 30 s, and 72°C for 45 s, and finally a final extension at 72°C for 10 min. PCR amplicons were size-selected on 2% agarose gels and purified using the AxyPrep DNA Gel Extraction Kit (Axygen Biosciences). After quantification using the Qubit 3.0 Fluorometer (Invitrogen), equimolar concentrations of uniquely barcoded amplicons (24 samples per pool) were combined for library preparation. The pooled amplicons were processed into an Illumina-compatible paired-end library following the manufacturer’s genomic DNA library preparation protocol. Final sequencing was performed on an Illumina MiSeq platform (2 × 250 bp chemistry; Shanghai BIOZERON Co., Ltd, Shanghai, China).

### 2.6 Processing of sequencing data

Raw paired-end sequences obtained from Illumina MiSeq sequencing underwent rigorous quality control and processing. Initial processing included: (1) read merging using FLASH v1.2.11 with default parameters, followed by (2) quality trimming with Trimmomatic v0.36 (sliding window: 4 bp; minimum quality score: 20) (Magoč and Salzberg, 2011; Bolger et al., 2014). Chimeric sequences were identified and removed using UCHIME v8.1 through *de novo* detection (Edgar et al., 2011). High-quality sequences were clustered into operational taxonomic units (OTUs) at a 97% similarity threshold using the UPARSE pipeline (v7.0.1090), with singletons excluded from downstream analysis (Edgar, 2013). Taxonomic classification was performed using the RDP Classifier v2.2 with Bayesian algorithm (confidence threshold: 0.8), utilizing the following reference databases: (1) SILVA release 138 for bacterial 16S rRNA gene sequences, and (2) UNITE v8.2 for fungal ITS region annotation (Kõljalg et al., 2005; Quast et al., 2012). After taxonomic classification, OTUs were filtered with the “filter_pollution()” function using “microeco package” in R (v.4.2.1) to remove mitochondrial and chloroplast sequences for subsequent microbial diversity analysis.

### 2.7 Bioinformatics and statistical analyses

Microbial community analyses were performed using QIIME 2 to calculate alpha diversity indices (Chao1, ACE, Shannon, and Simpson) and beta diversity based on Bray-Curti’s dissimilarity matrices (Bolyen et al., 2019) (Yang et al., 2023b). Significant differences in alpha diversity among treatments were assessed using Duncan’s multiple range test (P < 0.05) implemented in the “multcomp” package (R v.4.2.1). Beta diversity patterns were statistically evaluated through permutational multivariate analysis of variance (PERMANOVA) using the adonis() function (“vegan” package, 999 permutations) in R (Anderson and Walsh, 2013). Visualization of results was achieved using R packages: boxplots for alpha diversity indices (“ggplot2”), principal coordinate analysis (PCoA) plots for beta diversity (“vegan”), and relative abundance bar plots at phylum/order levels using an R script (Ahmed et al., 2022). Rarefaction curves at the OTU level were generated to assess the adequacy of sequencing depth. Differential abundance analysis at the genus level was performed using Tukey’s HSD test (P < 0.05, “multcomp” package), with results displayed as heatmaps of the top 30 species (“ggplot2”). For biomarker identification, we conducted linear discriminant analysis effect size (LEfSe) (Han et al., 2020) with a two-stage approach: (1) Kruskal-Wallis sum-rank test (P < 0.05) to detect significant abundance differences, followed by (2) linear discriminant analysis (LDA score > 2.5) to estimate effect sizes of differentially abundant taxa (Ijaz et al., 2018).

### 2.8 Isolation of the endophytes

According to the data analyses, key endophytic bacteria were isolated from non-inoculated regions of symptomatic leaves in the JJT (Cuimi + *Xcc*) treatment. The citrus leaves reserved for the isolation experiment were first rinsed with tap water, and diseased portions were removed. Next, surface sterilization was performed according to the procedure described above. After that, 5 mL of sterile water was added to a sterile mortar, and the leaves (approximately 0.5g) were processed into a homogenate, which was then kept at 28°C and 160 rpm for 30 minutes. The resulting slurry was collected and diluted into three different concentration gradients: 10^−2^, 10^−3^, and 10^−4^. A 100 μL sample from each dilution was spread onto King’s B (KB) medium and incubated at 28°C for 48 hours. After incubation, individual colonies with distinct morphological characteristics were picked from the plates. The strains were purified using the streak-plate technique, assigned numbers, and preserved at −20°C for later use.

### 2.9 Assessment of antibacterial activity of isolated bacterial strains against *Xcc*

The antibacterial activity of the isolated bacterial strains was evaluated against the *Xcc* strain 306. This well-characterized reference strain was selected for the *in vitro* assay to facilitate standardization and future comparisons with other studies. The assay was performed using the Oxford cup method. Initially, a 100 μL aliquot of strain 306 suspension (OD_600nm_ = 0.5; 108 CFU/mL) was spread evenly onto NA medium Petri plates. Subsequently, 100 μL of a culture of each isolated bacterial strain (OD_600nm_ = 0.8; 10^^8^ CFU/mL) was added to the Oxford cups, while NB medium served as the negative control. The plates were incubated at 28°C for 48 h in a constant-temperature incubator. After incubation, the diameter of the inhibition zones was measured.

### 2.10 Identification and characterization of potent endophytic bacterial strain

#### 2.10.1 Physiological and biochemical characterization

The bacterial strain exhibiting the most potent antibacterial activity was streaked onto KB agar and incubated at 28°C for 48 h to record morphological characteristics. Colony morphology, including color, texture (e.g., mucoid, dry, or smooth), surface appearance (shiny or dull), elevation, and margin (entire, undulate, or filamentous), was recorded. A single purified colony of the strain was streaked onto Biolog universal growth (BUG) media and incubated at 28°C for 24 h. The turbidimeter was zeroed using a sterile inoculating fluid, after which bacterial cells were collected from the colony surface with a sterile cotton swab and suspended in the inoculating fluid. The bacterial suspension was thoroughly mixed and adjusted to 90-98% turbidity. Subsequently, aliquots (100 µL) of the standardized suspension were dispensed into each well of a Biolog GENIII microplate. The plate was placed in a humidity chamber with moistened gauze and incubated at 28°C for 48 h. Phenotypic characterization was performed using the Microstation™ V4.01 (Biolog, Inc.).

#### 2.10.2 Molecular identification

The genomic DNA of the antagonistic strain was extracted using a bacterial genome extraction kit (Tiangen Biotech). The 16S rDNA region was amplified via PCR using universal primers 27F (5′-AGAGTTTGATCCTGGCTCAG-3′) and 1492R (5′-TACGGYTACCTTGTTACGACTT-3′) (Zhang et al., 2022). The PCR products were purified and sent to Qingke Biotechnology Co., Ltd. for Sanger sequencing. The obtained sequences were compared with the NT database at NCBI using BLASTn. Phylogenetic identification was performed using MEGA 12 with the Maximum-Likelihood method, and clade support was assessed through 1000 bootstrap replicates (Qiu et al., 2023). The primer pair phzNO1-F1 (5′-GTCGGAAGAAGAACGCCAGA-3′) and phzNO1-R1 (5′-ATAGTCGTTGGTGCAGACCG-3′) (Zhao et al., 2016) was used to amplify a 229-bp fragment specific to *Lysobacter antibioticus*. PCR reaction (25 μL) was conducted three times, containing 0.5 μL genomic DNA (50 ng/μL), 0.5 μL of each primer (10 μM/μL), 12 μL 2× EasyTaq PCR Supermix (TaKaRa), and 11.5 μL ddH_2_O. Amplification was performed with initial denaturation at 94°C for 30 s, followed by 30 cycles of 94°C for 30 s, 52°C for 30 s, and 72°C for 30 s, with a final extension at 72°C for 5 min. PCR products were electrophoresed at 4 V/cm on 1.2% agarose gels stained with SYBR Green I (10,000×; Solarbio Life Sciences) and visualized under UV light.

## 3 Results

### 3.1 Disease symptom assessment

The differential resistance of the two citrus cultivars to Xanthomonas citri subsp. citri (*Xcc*) was unequivocally demonstrated by the distinct symptomologies observed at three days post-inoculation (dpi; **Supplementary Figure S1**). In the susceptible ‘Orah’ cultivar (WGT treatment), Xcc inoculation resulted in severe disease symptoms characteristic of citrus canker. Infiltration sites developed into large, raised lesions with pronounced chlorosis (**Supplementary Figure S1A**). In contrast, the tolerant ‘Cuimi’ (JJT treatment) exhibited a markedly reduced symptomatic response. Infiltration sites developed only unraised and water-soaking lesions (**Supplementary Figure S1B**). Mock-inoculated control leaves for both ‘Orah’ (**Supplementary Figure S1C**) and ‘Cuimi’ (**Supplementary Figure S1D**) remained completely asymptomatic, confirming that the developed symptoms were solely due to Xcc infection.

### 3.2 Sequencing and assembly

A total of 12 leaf samples were analyzed using the Illumina MiSeq platform for 16S and ITS amplicon sequencing, yielding 454,199 (average: 37,850 per sample) and 460,564 (average: 38,380 per sample) sequences, with average lengths of 378 bp and 255 bp, respectively (**Supplementary Table S1**). The sequences were clustered into operational taxonomic units (OTUs) at 97% similarity for taxonomic classification, resulting in a total of 1272 bacterial and 775 fungal OTUs across the entire dataset. The sequencing data demonstrated validity and reliability for microbial diversity analysis. Rarefaction curves generated from the 12 samples confirmed that a sufficient number of reads were obtained to assess bacterial and fungal richness under all treatment conditions, indicating robust coverage of microbial communities (**Supplementary Figure S2**).

### 3.3 Alpha diversity analysis of endophytic microbial community

**Figure 1:**
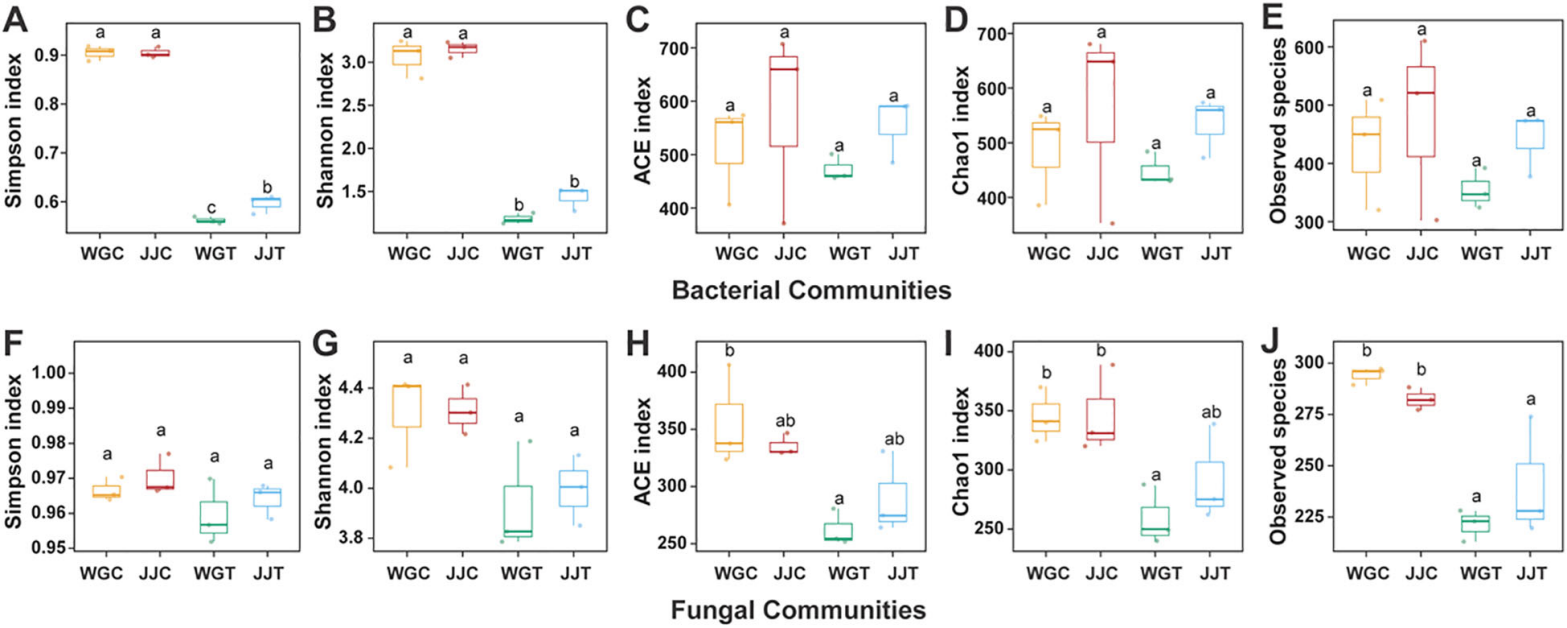
Alpha diversity of leaf endophytic bacterial and fungal communities in resistant (Cuimi) and susceptible (Orah) citrus cultivars following inoculation with Xanthomonas citri subsp. citri (Xcc). Boxplots showing within-sample diversity indices for bacterial **(A–E)** and fungal **(F–J)** communities. Treatments are: WGC (C. reticulata ‘Orah’ control), JJC (F. crassifolia ‘Cuimi’ control), WGT (Orah + Xcc), JJT (Cuimi + Xcc). **(A, F)** Simpson index. **(B, G)** Shannon index. **(C, H)** ACE index. **(D, I)** Chao1 index. **(E, J)** Observed species. Different lowercase letters above boxes indicate statistically significant differences among treatments, as determined by Duncan’s multiple range test (P < 0.05).

The within-sample diversity (α-diversity) of microbial communities was evaluated using Chao1, ACE, Shannon, and Simpson indices at a 97% similarity threshold across different treatments (**Figure 1**; **Supplementary Table S2**). For bacterial communities, the Shannon and Simpson indices were significantly lower in non-inoculated treatments compared to those inoculated with Xcc (WGT and JJT). Notably, the susceptible variety (WGT) exhibited a more pronounced decline in these values than the tolerant variety (JJT), with a significant difference observed in the Simpson index (**Figure 1**). In contrast, no significant differences were detected in Chao1 and ACE indices among treatments. Fungal communities displayed a distinct trend in α-diversity. Unlike bacteria, neither the Shannon nor the Simpson indices differed significantly across treatments (**Figure 1**). However, the Chao1 and ACE indices were considerably higher in non-inoculated controls (WGC and JJC) than in Xcc-inoculated treatments (WGT and JJT). Overall, the differences in endophytic bacterial communities were primarily driven by diversity metrics (Shannon and Simpson indices), whereas variations in fungal communities were mainly associated with richness indices (Chao1 and ACE).

### 3.4 Beta diversity analysis of endophytic microbial community

**Figure 2:**
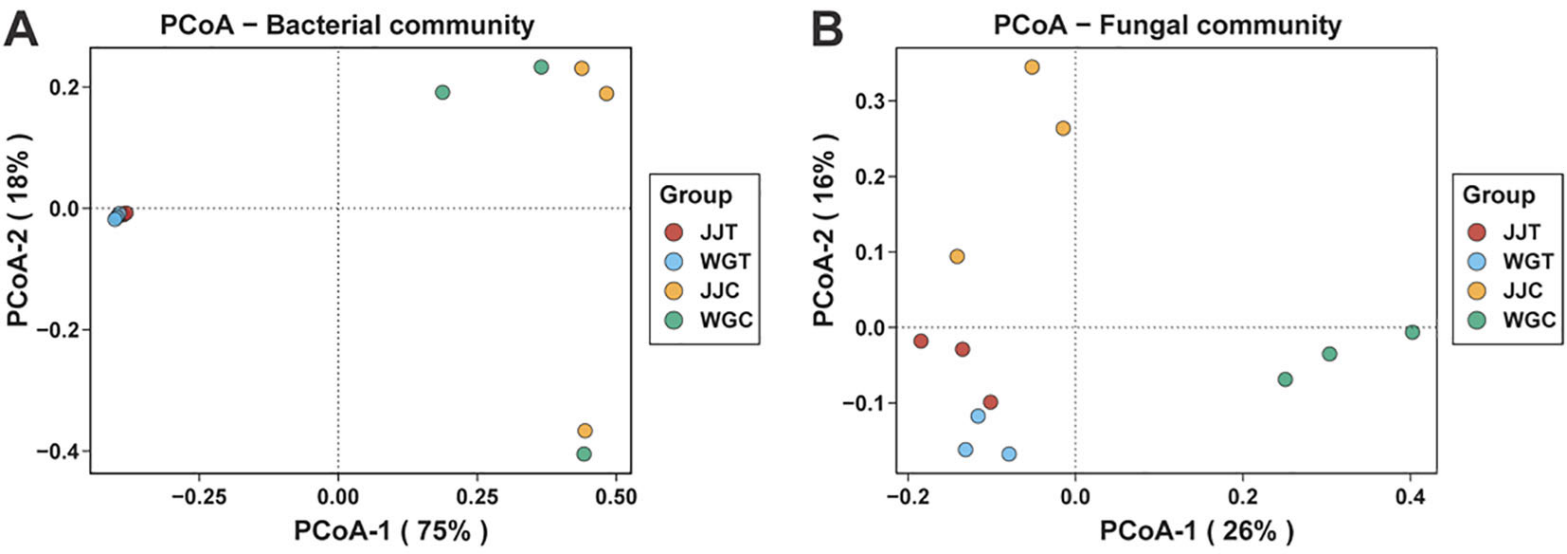
Beta diversity of leaf endophytic microbial communities in response to Xcc infection. Principal coordinate analysis (PCoA) plots based on Bray-Curtis dissimilarity, illustrating the structural differences in bacterial **(A)** and fungal **(B)** community composition among treatments. Treatments are: WGC (C. reticulata cv. ‘Orah’ control), JJC (F. crassifolia cv. ‘Cuimi’ control), WGT (Orah + Xcc), and JJT (Cuimi + Xcc).

Principal coordinate analysis (PCoA) based on Bray-Curti’s dissimilarity was used to assess structural shifts in bacterial and fungal communities across treatments (**Figure 2**; **Supplementary Table S3**). The first two PCoA axes explained 75% (PC1) and 18% (PC2) of the variation in bacterial communities (**Figure 2A**), and 26% (PC1) and 16% (PC2) in fungal communities (**Figure 2B**). For bacterial communities, inoculated (Xcc-exposed: WGT, JJT) and non-inoculated (WGC, JJC) treatments showed clear separation (**Figure 2A**). The high variance explained by PC1 revealed subtle distinctions between WGC and JJC, as well as between WGT and JJT. Fungal communities exhibited pronounced dispersion across all treatments (**Figure 2B**). Specifically, WGT diverged from WGC along PC1, while JJT separated from JJC along PC2. PERMANOVA confirmed significant treatment effects on both bacterial (R² = 0.757, P < 0.01) and fungal (R² = 0.495, P < 0.001) community structures (**Supplementary Table S3**). These results demonstrate that Xcc invasion significantly alters the diversity and composition of endophytic microbiomes. Notably, bacterial and fungal communities responded differently, consistent with alpha diversity trends, suggesting distinct ecological adaptation mechanisms.

### 3.5 Differential abundance and community composition at the phylum and order level

**Figure 3:**
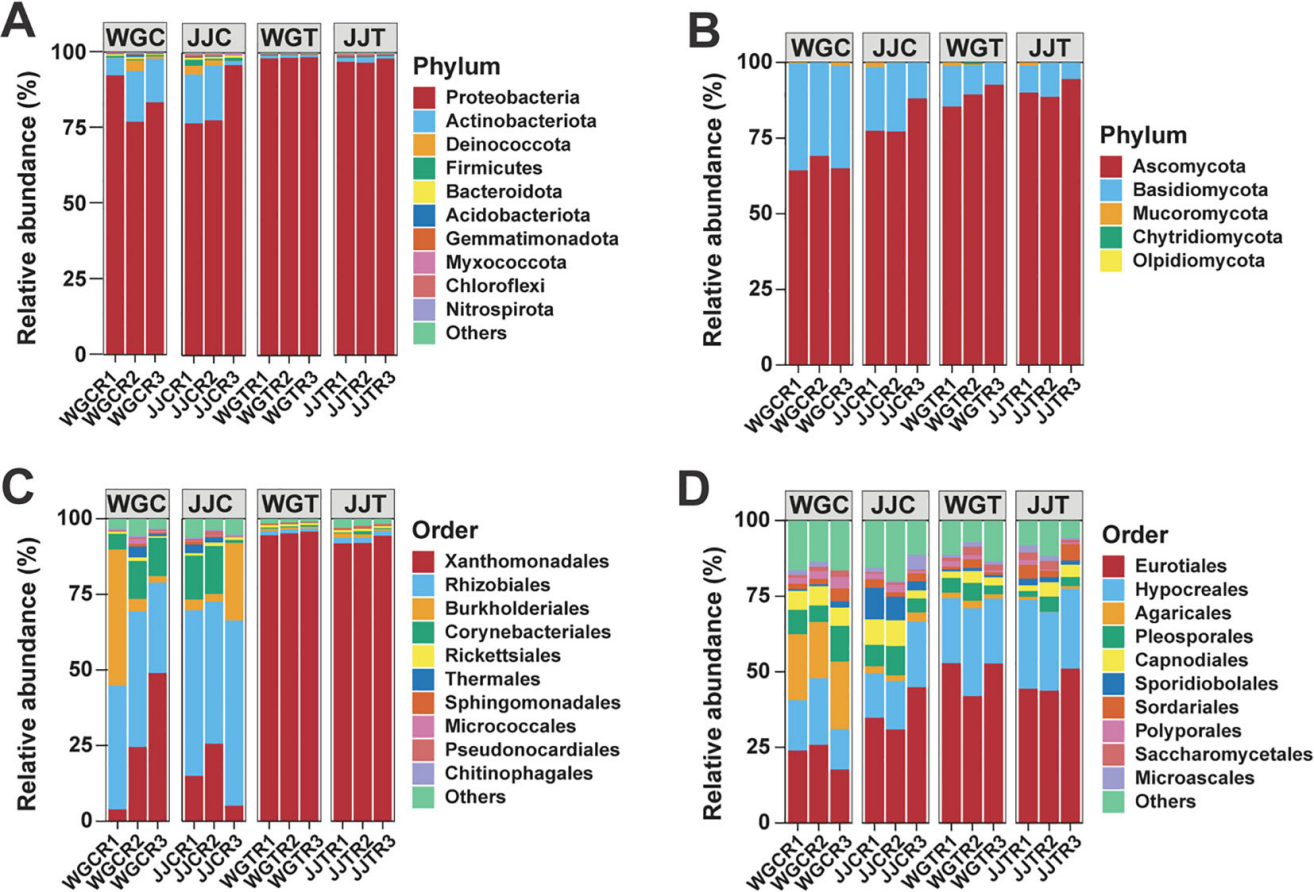
Taxonomic composition of leaf endophytic bacterial and fungal communities. Bar plots show the relative abundance of the top 10 most abundant bacterial **(A)** and fungal **(B)** phyla, and bacterial **(C)** and fungal **(D)** orders across the different treatments. Treatments are: WGC (C. reticulata cv. ‘Orah’ control), JJC (F. crassifolia cv. ‘Cuimi’ control), WGT (Orah + Xcc), and JJT (Cuimi + Xcc). All other taxa are summarized in the “Others” category. The total relative abundance of the presented taxa is indicated for each panel.

The overall structure of the endophytic communities at the phylum and order levels is shown in **Figure 3**. Phyla such as Proteobacteria and Actinobacteria dominated bacterial communities, while fungal communities were primarily composed of Ascomycota and Basidiomycota (**Figures 3A, B**; **Supplementary Table S4**). At the order level, Xanthomonadales, Rhizobiales, and Burkholderiales were the most abundant bacteria, and Eurotiales, Hypocreales, and Agaricales were the most abundant fungi (**Figures 3C, D**; **Supplementary Table S5**).

### 3.6 Key taxonomic differences identified by LEfSe

**Figure 4:**
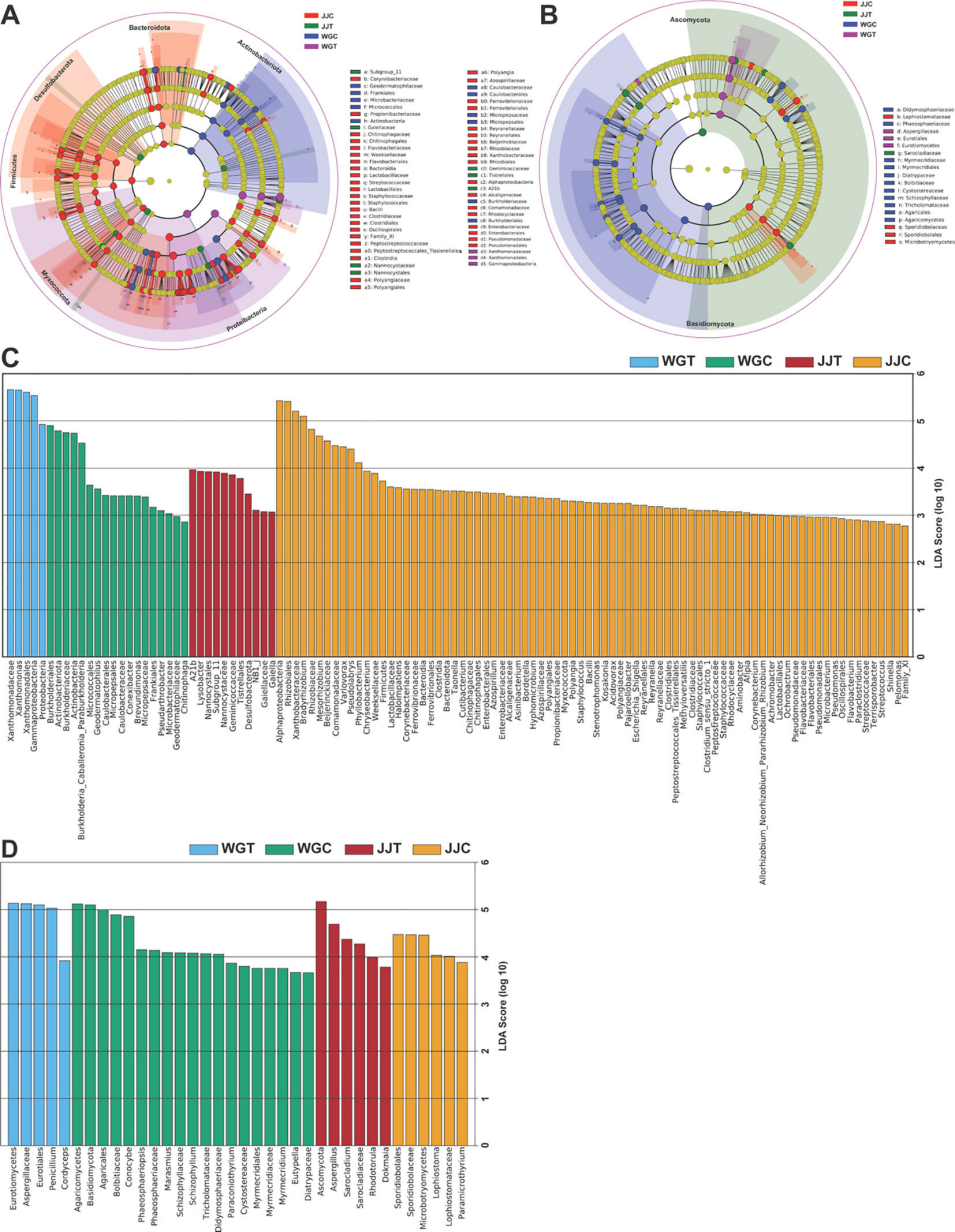
Identification of microbial biomarkers distinguishing treatment groups using LEfSe analysis. Cladograms **(A, B)** and histograms of Linear Discriminant Analysis (LDA) scores **(C, D)** for bacterial **(A, C)** and fungal **(B, D)** communities. The cladograms illustrate the phylogenetic distribution of significant taxa, where colored nodes indicate taxa that are statistically significant biomarkers in the corresponding colored treatment group. Gray nodes represent non-significant taxa. The LDA score histograms **(C, D)** illustrate the effect size of biomarkers with an LDA score greater than 2.5 (Kruskal-Wallis test, p < 0.05) for each treatment. Treatments are: WGC (*C. reticulata* cv. ‘Orah’ control), JJC (*F. crassifolia* cv. ‘Cuimi’ control), WGT (Orah + *Xcc*), and JJT (Cuimi + *Xcc*).

To identify the specific taxa that were statistically significant biomarkers of each treatment, we employed LEfSe analysis (Kruskal-Wallis sum-rank test, p < 0.05), with linear discriminant analysis (LDA > 2.5) quantifying effect sizes (**Figure 4**; **Supplementary Tables S6, S7**). Bacterial communities showed significant differences in 5 (WGT), 18 (WGC), 11 (JJT), and 80 (JJC) taxa (**Figure 4A**). In contrast, fungal communities exhibited differences in 5 (WGT), 19 (WGC), 6 (JJT), and 6 (JJC) taxa (LDA > 2.5, p < 0.05; (**Figure 4B**). These results demonstrate that Xcc invasion suppresses bacterial diversity in both tolerant and susceptible varieties, with the tolerant variety maintaining more biomarkers both before and after inoculation. In susceptible plants (WGT), Xcc enrichment was observed across multiple taxonomic levels (Proteobacteria, Gammaproteobacteria, Xanthomonadales, Xanthomonadaceae, and Xanthomonas) (**Figure 4C**; **Supplementary Table S6**). In contrast, the tolerant variety (JJT) was characterized by 11 biomarker taxa. Among these, the genus Lysobacter was identified as a key biomarker, and non-inoculated treatments were characterized by Burkholderiales (9 taxa in JJC; 3 in WGC). Fungal communities exhibited a distinct pattern. The susceptible variety (WGT) was characterized by a high-abundance consortium of biomarkers, including Penicillium and Aspergillus, whereas the tolerant variety (JJT) was primarily defined by Aspergillus alone (**Figure 4D**; **Supplementary Table S7**). Notably, the number of fungal biomarkers identified for the tolerant cultivar was consistent between its control (JJC) and inoculated (JJT) states (6 taxa each). In contrast, the susceptible cultivar showed a reduction in biomarkers post-inoculation (from 19 in WGC to 5 in WGT). This suggests a more resilient fungal community structure in the tolerant cultivar, which maintained a consistent level of taxonomic distinction despite the significant community shifts confirmed by beta diversity analysis.

### 3.7 Key microbial genera differentiate treatment responses

**Figure 5:**
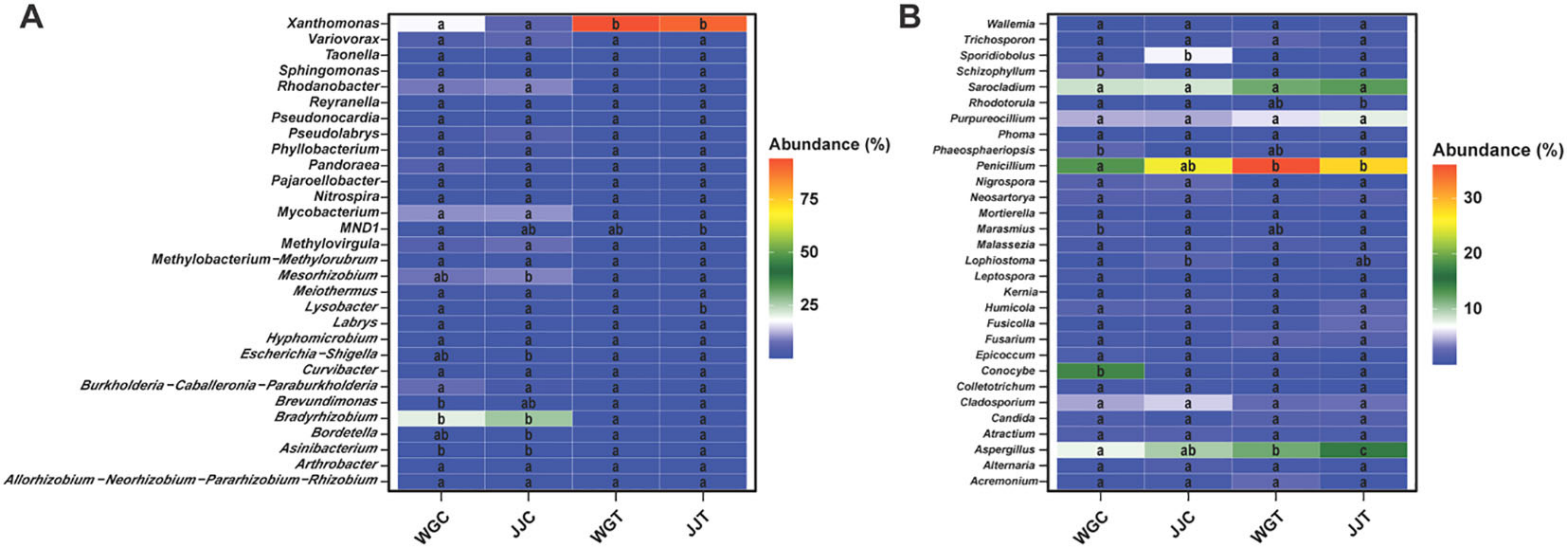
Heatmap analysis of dominant endophytic bacterial and fungal genera. The relative abundance (Z-score scaled) of the top 30 most abundant bacterial **(A)** and fungal **(B)** genera across the different treatments. Treatments are: WGC (C. reticulata cv. ‘Orah’ control), JJC (F. crassifolia cv. ‘Cuimi’ control), WGT (Orah + Xcc), and JJT (Cuimi + Xcc). The color gradient from blue to red indicates low to high relative abundance, respectively. Genera showing significant differences in abundance among treatments (Tukey’s HSD test, p < 0.05) are included.

The relative abundance patterns of the 30 most dominant genera further illustrated the community-wide reshuffling induced by Xcc invasion (**Figure 5**; **Supplementary Tables S6, S7**). The top 30 bacterial genera represented 94.66% of the total relative abundance (RA), with Xanthomonas (51.94%) and Bradyrhizobium (12.17%) being the most dominant (**Supplementary Table S8**). The heatmap visualization confirmed the LEfSe results, showing a massive increase in Xanthomonas RA in inoculated plants and a notable increase in Lysobacter in the tolerant JJT treatment (**Figure 5A**; **Supplementary Table S8**). Furthermore, it revealed a dramatic suppression of putative beneficial genera, such as Bradyrhizobium, Asinibacterium, and Mesorhizobium, in both cultivars following inoculation. A similar restructuring was observed in the fungal community. LEfSe identified Penicillium and Aspergillus as key biomarkers for the susceptible inoculated cultivar (WGT), while only Aspergillus was a significant biomarker for the tolerant inoculated plants (JJT). The heatmap of the top 30 fungal genera (representing 83.09% of total RA) confirmed these patterns, showing a cultivar-dependent response for Penicillium, which surged in the susceptible cultivar ‘Orah’ (WGT) but remained stable in the tolerant ‘Cuimi’ (JJT) (**Figure 5B**; **Supplementary Table S9**). In contrast, Aspergillus RA increased significantly in both cultivars under pathogen pressure. Notably, 26 of the 30 dominant fungal genera are classified as saprophytes or secondary pathogens, suggesting their proliferation may accelerate tissue senescence and indirectly promote canker progression. Collectively, these analyses demonstrate that Xcc invasion markedly reshapes endophytic community composition. The distinct, cultivar-specific responses of key genera, such as Lysobacter and Penicillium, as identified by LEfSe, underscore their potential critical roles in host-microbe interactions during pathogen challenges.

### 3.8 Screening of potent antibacterial strain

**Figure 6:**
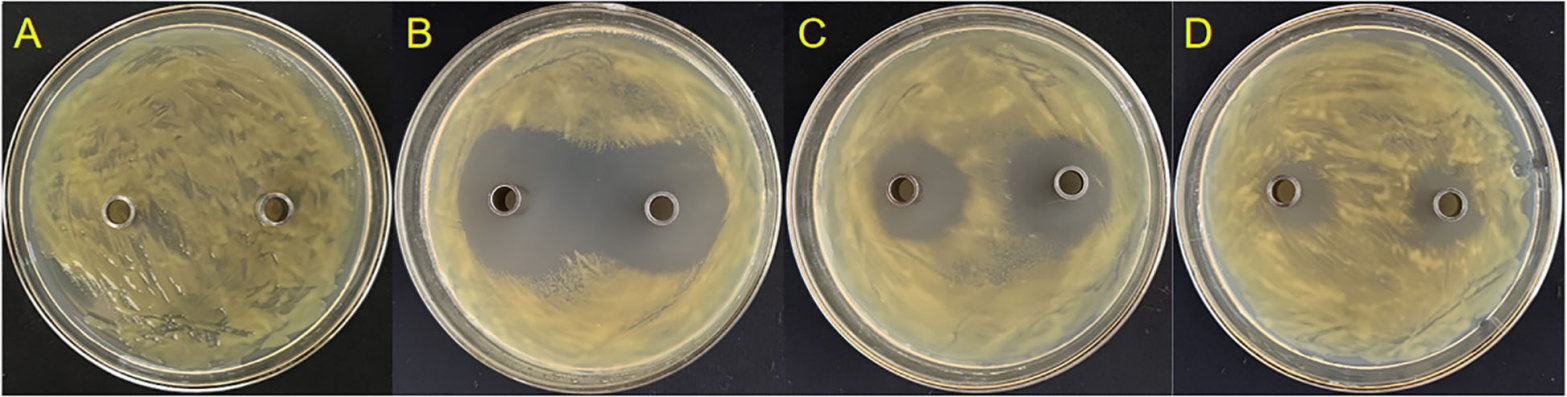
*In vitro* antagonistic activity of endophytic bacterial isolates against Xcc. **(A)** Negative control (CK) showing no inhibition zone. **(B)** Strain GJ-6 exhibited a potent and clear inhibition zone (37.6 ± 0.1 mm in diameter). **(C)** Strain GJ-11 shows a smaller inhibition zone. **(D)** Strain GJ-14 shows weak or no antagonistic activity. The antibacterial activity was assessed using the Oxford cup method on nutrient agar plates. The diameter of the clear zone around the cup indicates the strength of antagonism against the lawn of Xcc strain 306.

Three endophytic bacteria, suspected to be Lysobacter strains, were successfully isolated and purified from stored leaf samples. Strain GJ-6 exhibited the most potent antagonistic activity against Xcc. Bioassay results demonstrated that the original concentration of GJ-6 fermentation broth produced a pronounced inhibition zone, measuring 37.6 ± 0.1 mm (± SD), against Xcc, indicating superior antibacterial efficacy compared to other isolates (GJ-11 and GJ-14) (**Figure 6**).

### 3.9 Identification and characterization of the biocontrol strain Lysobacter antibioticus GJ-6

#### 3.9.1 Physiological and biochemical characteristics of GJ-6

**Figure 7:**
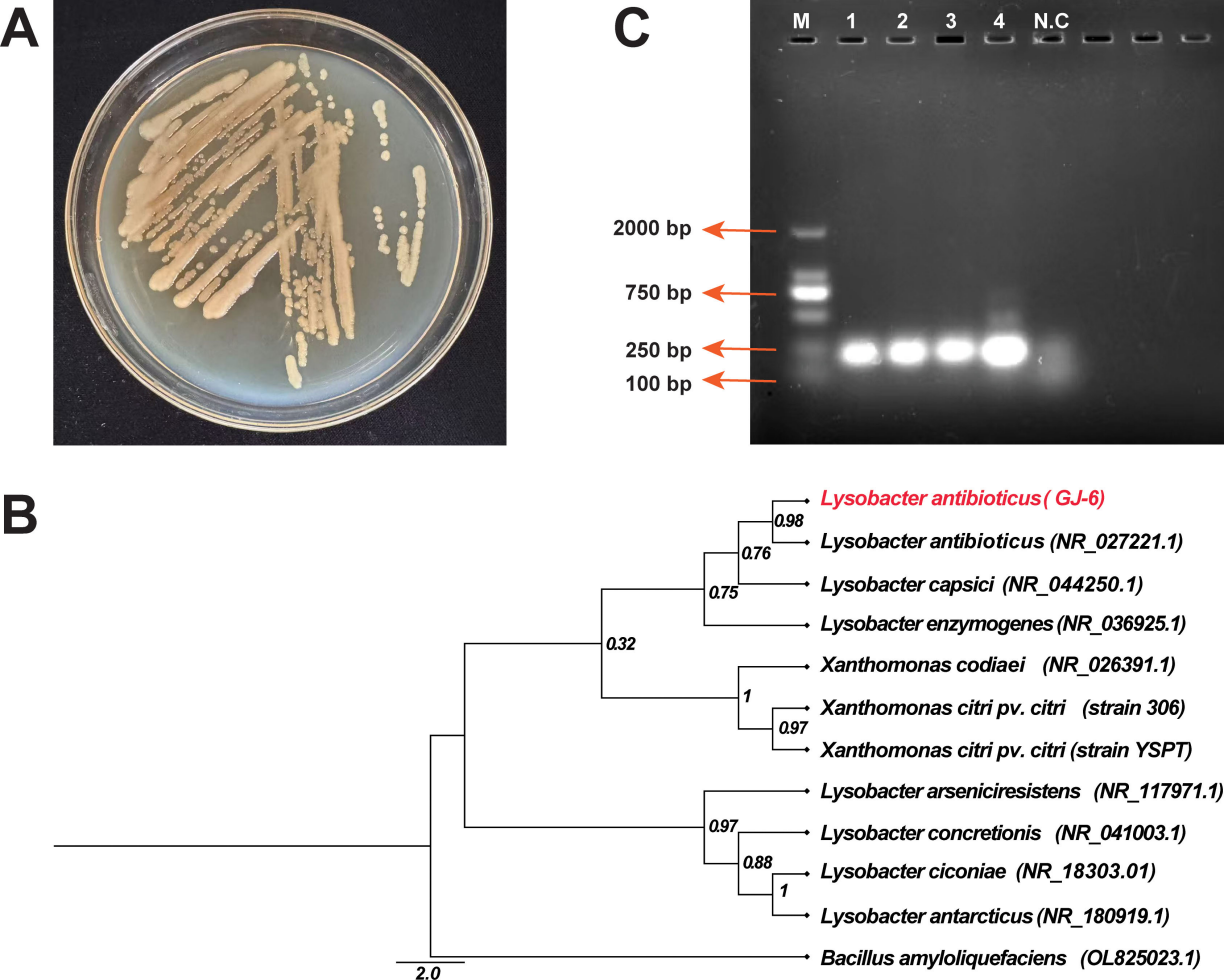
Identification and characterization of the biocontrol strain Lysobacter antibioticus GJ-6. **(A)** Colony morphology of strain GJ-6 after 48 hours of growth on King’s B agar medium. **(B)** Maximum-Likelihood phylogenetic tree based on 16S rRNA gene sequences, showing the relationship of strain GJ-6 (highlighted) to closely related Lysobacter type strains. Bootstrap values (based on 1000 replicates) are shown at the nodes. The scale bar indicates the number of nucleotide substitutions per site. **(C)** Molecular confirmation of L. antibioticus GJ-6 using species-specific phzNO1primers. Here, M = 2000 bp marker, 1-4 = PCR product of 229 bp containing genomic DNA of strain GJ-6, and N.C = Negative control (without DNA).

After 48 hours on KB medium, strain GJ-6 formed round, light-yellow, smooth, moist, mucoid colonies with a shiny surface, central elevation, and entire margins. With further incubation, their color gradually intensified (**Figure 7A**). Ninety-four phenotypic traits (71 carbon/nitrogen utilization and 23 chemical sensitivity tests) were evaluated using the Biolog platform (**Supplementary Table S10**). The results demonstrated that GJ-6 could actively utilize 30 substrates, including D-maltose, D-trehalose, α-D-lactose, D-mannose, D-fructose, D-glucose-6-phosphate, gelatin, L-arginine, L-aspartic acid, L-glutamic acid, citric acid, L-malic acid, and acetic acid. In contrast, it showed variable utilization of 35 other substrates and was unable to metabolize six substrates, including stachyose, inosine, D-sorbitol, D-serine, p-hydroxyphenylacetic acid, and α-ketoisobutyric acid. Among the 23 chemical sensitivity tests, GJ-6 showed no inhibition in the presence of 11 agents or conditions (e.g., pH 6, 1% NaCl, 1% sodium lactate, rifamycin SV, lincomycin, vancomycin, potassium tellurite, and aztreonam). However, it was clearly inhibited by seven agents (including pH 5, 4% NaCl, fusidic acid, minocycline, Niaproof 4, and sodium bromate) and exhibited borderline sensitivity to the remaining five. These metabolic features are consistent with the known characteristics of the genus Lysobacter.

#### 3.9.2 Molecular characteristics of GJ-6

Molecular identification via 16S rDNA sequencing (amplified 1370 bp fragment using primers 27F/1492R) revealed 99.85% similarity to Lysobacter antibioticus (NR_027221.1) in the NCBI database, with phylogenetic analysis further confirming L. antibioticus as the closest relative (**Figure 7B**). The 16S rRNA-based phylogeny delineated three clusters: one comprising plant-associated species (L. antibioticus, L. capsici, and L. enzymogenes), all known as biocontrol agents; a distinct clade containing the pathogen Xcc, clearly separated from GJ-6; and a third cluster of environmentally derived Lysobacter species (L. antarcticus, L. ciconiae, and L. arseniciresistens). Strain-specific PCR using phzNO1 primers amplified a 229 bp fragment (**Figure 7C**), confirming phenazine biosynthesis genes in GJ-6 and correlating with its antimicrobial activity against Xcc, while also distinguishing L. antibioticus from related species (L. capsici, L. enzymogenes, etc.). Together, these results morphological, metabolic, phylogenetic, and genetic definitively identify strain GJ-6 as Lysobacter antibioticus and support its potential as a biocontrol agent.

## 4 Discussion

Citrus is a globally vital crop, with its production in China’s Guangxi Province being particularly significant to the agricultural economy. However, citrus cultivation is severely threatened by citrus canker, a devastating disease caused by *Xanthomonas citri* subsp. *citri* (*Xcc*) that leads to substantial economic losses (Fathi et al., 2024; Pena et al., 2024). While integrated management strategies exist, control still heavily relies on chemical pesticides, raising concerns about environmental impact, pathogen resistance, and cost. Despite the absence of complete genetic resistance in citrus, cultivars display a spectrum of susceptibility to *Xcc*, ranging from the highly susceptible ‘Orah’ mandarin to the partially resistant kumquat and calamondin (Chen et al., 2012; Sharma et al., 2022). This variation highlights an opportunity to explore alternative control strategies. In this context, endophytes, resident plant microbes, have emerged as promising, sustainable resources for disease management (Islam et al., 2019). For instance, studies on kiwifruit canker have successfully linked distinct endophytic bacterial communities to host resistance, leading to the isolation of potential biocontrol agents like *Lysobacter* and *Pseudomonas* (Fu et al., 2024; Zheng et al., 2024). Despite these advances in other pathosystems, the role of the endophytic microbiome in conferring resistance to citrus canker remains poorly understood. Preliminary work in citrus suggests a correlation between endophytic bacteria and resistance, identifying *Bacillus subtilis* as a candidate (Liu et al., 2021a). However, a comprehensive understanding is lacking, particularly regarding the response of the entire microbial community, including the often-overlooked fungal endophytes, across cultivars with defined resistance phenotypes.

Plant microbiome homeostasis is crucial for maintaining plant health and preventing dysbiosis, a state of microbial imbalance associated with adverse outcomes (Paasch and He, 2021; Pfeilmeier et al., 2021). This equilibrium is shaped by a complex interplay of host genetics, pathogen characteristics, and environmental factors (Chen et al., 2020; Entila et al., 2024; Su et al., 2024). In our study, we standardized environmental and inoculation conditions to specifically isolate the influence of cultivar resistance on endophytic microbial responses to *Xcc* challenge. Furthermore, we used specific 16S rDNA primers to minimize host organelle contamination, ensuring an accurate profile of the endophytic bacteriome (Anguita-Maeso et al., 2022). Our results revealed that *Xcc* invasion triggered distinct responses in the bacterial and fungal communities. For bacteria, pathogen challenge significantly reduced diversity (Shannon and Simpson indices), while leaving richness (Chao1 and ACE indices) largely unaffected. This suppression of bacterial diversity is supported by PCoA, which showed clear separation between infected and non-infected plants, and aligns with previous findings in citrus canker pathosystems (Zhou et al., 2023). However, we assumed that the direct infiltration of a high *Xcc* biomass might be the primary driver of the bacterial community separation observed along PC1 (75% of variance). Future long-term studies are needed to determine if this effect is due solely to the high inoculum of *Xcc* or to *Xcc* ability to actively alter the host bacteriome.

In contrast, the fungal community responded differently. Pathogen invasion primarily reduced community richness, as indicated by PcoA, which revealed a distinct restructuring post-inoculation. This divergent response between the bacterial and fungal kingdoms underscores their unique ecological roles and adaptation mechanisms during pathogen stress, a phenomenon observed in other pathosystems, such as maize stalk rot (Xia et al., 2024). An important consideration in interpreting the bacterial diversity results is the method of pathogen inoculation. The syringe infiltration technique, while ensuring synchronized and high-efficiency infection for comparative purposes, introduces a substantial and immediate biomass of *Xcc* directly into the apoplast. This artificial input drastically alters community evenness, a key component of diversity indices such as the Shannon and Simpson indices. Therefore, the pronounced reduction in these indices in inoculated plants is likely driven by a combination of the pathogen’s biological activity *and* its direct physical introduction. While this method effectively standardizes infection and reveals how a massive pathogen challenge disrupts a resident microbiome, it does not fully recapitulate the more gradual, natural infection process initiated through stomata or wounds. Future studies aiming to investigate microbiome dynamics under conditions mimicking natural epidemics more closely would benefit from alternative methods, such as spray inoculation, which avoids the direct biomass confounder and allows for the observation of community shifts during the pathogen’s initial colonization and invasion phases.

Taxonomic composition analysis revealed distinct community structures for bacteria and fungi. The bacterial community was dominated by Proteobacteria (90.82%) and Actinobacteria (6.54%), primarily comprised of the orders Xanthomonadales (57.22%), Rhizobiales (23.97%), and Burkholderiales (7.34%). Fungal communities were primarily composed of Ascomycota (81.86%) and Basidiomycota (17.62%), with Eurotiales (38.73%), Hypocreales (21.52%), and Agaricales (6.45%) as the predominant orders. As expected, a marked increase in the relative abundance of *Xanthomonas* at the genus level directly confirmed successful *Xcc* colonization, validating previous culture-based findings (Teper et al., 2020). Our results revealed contrasting patterns, with genera often cited as key biocontrol agents against citrus canker (Sudyoung et al., 2020; Yang et al., 2023a). The relative abundance of *Bradyrhizobium* was dramatically reduced in the community profiles of both susceptible (from 20.55% to 0.53%) and tolerant (from 26.78% to 0.80%) cultivars following inoculation. However, future studies utilizing absolute quantification methods (e.g., qPCR) are needed to conclusively determine the population dynamics of *Bradyrhizobium* and other taxa during infection. Furthermore, other putative biocontrol genera like *Bacillus*, *Pseudomonas*, and *Streptomyces* were absent among the dominant taxa in our experimental setup, potentially due to the strong selective pressure of direct *Xcc* inoculation. Notably, *Lysobacter* emerged as the most prominent bacterial genus, exhibiting a significantly increased abundance in tolerant varieties under pathogen pressure. It is important to note that LEfSe identified a consortium of enriched taxa in the tolerant cultivar, with *Lysobacter* being the most notable at the genus level due to its well-established biocontrol function. This cultivar-specific response suggests a potential functional role in the resistance mechanism. This finding is strongly supported by well-documented evidence of *Lysobacter*’s biocontrol capabilities. For instance, both *L. antibioticus* and *L. gummosus* exhibit antagonism against xanthomonads (Expósito et al., 2015), and *L. antibioticus* is known to produce phenazine antibiotics, such as myxin, which have broad-spectrum activity against various *Xanthomonas* pathogens (Zhao et al., 2016). Beyond direct antagonism, *L. antibioticus* can also enhance plant disease resistance by modulating the associated microbiome (Chen et al., 2025). Collectively, this evidence positions *Lysobacter* as a critical defensive component in the tolerant citrus cultivar, likely operating through both direct pathogen inhibition and indirect plant-mediated protection.

Among the dominant fungal genera identified, a significant proportion (26 out of 30) are documented as saprophytes or pathogens associated with senescent tissues (Jayasekara et al., 2022; Huang et al., 2024). These opportunistic fungi, including *Penicillium*, *Aspergillus*, *Sarocladium*, *Purpureocillium*, and *Cladosporium*, exhibited distinct responses to *Xcc* infection. The most abundant genus, *Penicillium*, displayed a clear cultivar-dependent dynamic, with its relative abundance surging from 13.11% to 36.04% in the susceptible ‘Orah’ but remaining stable in the tolerant ‘Cuimi’ (25.14% to 27.95%). In contrast, *Aspergillus* increased significantly in both cultivars under pathogen pressure (Perrone et al., 2007; Grantina-Ievina et al., 2013). The differential behavior of these fungi highlights how host resistance modulates their proliferation during pathogen challenge. The well-documented roles of *Penicillium* species (e.g., *P. digitatum*, *P. italicum*) in causing postharvest rot (Bullerman, 2003), and *Aspergillus* (e.g., *A. niger*, *A. flavus*) as an opportunistic pathogen (Lugauskas and Stakeniene, 2002; Bamba and Sumbali, 2005; Marino et al., 2009), are consistent with our findings. We propose that these endophytic fungi are not merely present but likely exacerbate the severity of citrus canker. The cultivar-specific explosion of *Penicillium* in susceptible plants, coupled with the universal increase in *Aspergillus*, suggests they exploit the physiological weakness induced by *Xcc*. This likely accelerates leaf senescence and compromises tissue integrity, potentially creating a damaging feedback loop that facilitates further disease progression (Juybari et al., 2019; Nicoletti, 2019; Sadeghi et al., 2019). To identify microbial taxa associated with each treatment, we performed LEfSe analysis, which revealed distinct biomarker signatures (Liang et al., 2023). *Xanthomonas*, *Penicillium*, and *Aspergillus* were identified as key biomarkers for the susceptible cultivar under disease pressure. At the same time, *Lysobacter* and *Aspergillus* were identified as biomarkers for the resistant, inoculated cultivar, whereas the non-inoculated controls were characterized by Burkholderiales. This analysis provides a robust, two-dimensional validation of our findings: *Penicillium* is highlighted as a primary fungal indicator of susceptibility in ‘Orah’, whereas *Lysobacter* is strongly associated with the resistance of ‘Cuimi’ (Han et al., 2020; Wei et al., 2022). The concurrent enrichment of *Aspergillus* as a biomarker in both cultivars, albeit represented by distinct OTUs, suggests a universal stress response to Xcc infection within this genus. This indicates that different *Aspergillus* lineages, potentially with varying saprophytic or opportunistic pathogenic capabilities, proliferate in the distinct physiological environments of susceptible and tolerant hosts (Huang et al., 2023).

The biocontrol efficacy of *L. antibioticus* is underpinned by a diverse arsenal of antagonistic mechanisms. Beyond the production of well-characterized phenazine antibiotics, this genus also synthesizes other bioactive metabolites, such as p-aminobenzoic acid, which disrupts pathogenicity in *Xanthomonas* by compromising membrane integrity, inhibiting motility, and suppressing biofilm formation (Jiang et al., 2023). Our previous work has systematically detailed a multi-target mode of action for its phenazines, which includes disrupting quorum sensing, inhibiting biofilm and EPS production, impairing flagellar synthesis, and compromising cell membrane integrity (Liu et al., 2022). The optimization of fermentation processes has further enhanced the production of these inhibitory compounds, collectively establishing *L. antibioticus* strains as potent agents capable of suppressing pathogens through direct antimicrobial activity and virulence attenuation (Liu et al., 2021c). Successful biocontrol ultimately relies on a bacterium’s ability to achieve three key objectives: niche colonization, antimicrobial production, and/or induction of systemic resistance in the host (Bonaterra et al., 2022). Among these, the capacity to establish robust colonization within host tissues is a critical first step (Compant et al., 2010; Maurer et al., 2013). Therefore, our strategy of screening the native citrus endosphere for antagonistic strains, such as *L. antibioticus* GJ-6, is optimal for sustainable canker management (Poveda et al., 2021). This approach leverages microbes that are pre-adapted to the citrus environment, thereby increasing the likelihood of successful establishment and pathogen suppression while ensuring ecological compatibility. While our study provides strong correlative evidence and isolates a potent antagonistic strain, *L. antibioticus* GJ-6, a causal relationship between its in planta enrichment and the host’s resistance phenotype requires further validation. The logical next step to bridge this gap is a re-introduction experiment, where the susceptible ‘Orah’ cultivar is inoculated with strain GJ-6 and then challenged with *Xcc*. Determining whether GJ-6 can colonize the host and reduce disease severity to recapitulate a resistant phenotype is essential. Such a study, coupled with investigations into its colonization efficiency and potential to induce systemic resistance, will be critical to definitively establish causation and thoroughly evaluate the biocontrol potential of *L. antibioticus* GJ-6 against citrus canker.

## 5 Conclusions

This study provides valuable insights into the differential responses of endophytic microbial communities in citrus cultivars with varying resistance to *Xanthomonas citri* subsp. *citri* infection. Our findings reveal that the pathogen significantly alters the microbial diversity in susceptible cultivars, particularly affecting bacterial and fungal communities. In contrast, tolerant cultivars exhibited less severe disruption to their microbial communities, characterized by the maintenance of bacterial diversity, including specific taxa such as *Lysobacter*, and a more restrained proliferation of opportunistic fungi under pathogen pressure. These shifts, observed in the context of a significantly stronger resistance phenotype in Cuimi, suggest that *Lysobacter* may play a pivotal role in the defense mechanisms of resistant citrus cultivars. Moreover, the study highlights the importance of endophytic microbiomes in influencing plant health and promoting pathogen resistance. The differential response of bacterial and fungal communities to *Xcc* infection underscores the complexity of host-microbe interactions and the potential of microbiome-based strategies for disease management. The identification of key microbial taxa associated with resistance provides a promising foundation for future research aimed at enhancing citrus resistance to *X. citri* through microbiome engineering or targeted biocontrol approaches. In conclusion, the results suggest that leveraging the native microbial communities in citrus could offer a sustainable alternative to chemical control methods for managing citrus canker. Further exploration of the identified taxa and their biocontrol mechanisms will be essential for developing effective, environmentally friendly strategies to combat this devastating disease.

## Data Availability

All the raw 16S and ITS sequence data can be found in the National Genomics Data Center (NGDC) BioProject database with the accession numbers PRJCA047478 and PRJCA047530.
